# Evolutionary predictions for a parasite metapopulation: Modelling salmon louse resistance to pest controls in aquaculture

**DOI:** 10.1111/eva.13618

**Published:** 2023-11-23

**Authors:** Andrew Coates, Nicholas A. Robinson, Tim Dempster, Ingrid Johnsen, Ben L. Phillips

**Affiliations:** ^1^ Sustainable Aquaculture Laboratory – Temperate and Tropical (SALTT), Queenscliff Marine Science Centre Deakin University Burwood Victoria Australia; ^2^ Breeding and Genetics, Nofima Ås Norway; ^3^ Institute of Marine Research Bergen Norway; ^4^ School of Molecular and Life Sciences Curtin University Bentley Western Australia Australia

**Keywords:** evolution model, integrated pest management, meta‐population, pesticide resistance, sea lice

## Abstract

Pests often evolve resistance to pest controls used in agriculture and aquaculture. The rate of pest adaptation is influenced by the type of control, the selective pressure it imposes, and the gene flow between farms. By understanding how these factors influence evolution at the metapopulation level, pest management strategies that prevent resistance from evolving can be developed. We developed a model for the metapopulation and evolutionary dynamics of the salmon louse (*Lepeophtheirus salmonis*), which is a major parasite affecting salmon aquaculture. Different management scenarios were simulated across a network of salmon farms covering half of Norway, and their effects on louse epidemiology and evolution were investigated. We compared louse controls that differed in how they were deployed through time (discrete vs. continuous), how they impacted the louse life cycle, and in their overall efficacy. We adjusted the strength of selection imposed by treatments, the dominance effect of the resistant allele, and the geographic location at which resistance originated. Continuously acting strategies (e.g., louse‐resistant salmon) were generally more effective than discrete strategies at controlling lice, especially when they increased louse mortality during early developmental stages. However, effective strategies also risked imposing frequent and/or strong selection on lice, thus driving rapid adaptation. Resistant alleles were more likely to be lost through genetic drift when they were recessive, had a low‐fitness advantage, or originated in low‐farm‐density areas. The north‐flowing current along the Norwegian coastline dispersed resistant genes from south to north, and limited gene flow in the opposite direction. We demonstrate how evolutionary models can produce quantitative predictions over large spatial and temporal scales and for a range of pest control scenarios. Quantitative outputs can be translated into practical management decisions applied at a regional level to minimise the risk of resistance developing.

## 1 INTRODUCTION

A major obstacle to sustainable pest management in agriculture is the evolution of treatment resistance by parasites and pathogens (Gould et al., 2018). Integrated pest management – using strategies such as pesticide rotation and refugia – can slow or halt the evolution of resistance (Carrière et al., 2010; McEwan et al., 2016; Rimbaud et al., 2018). Integrated pest management introduces additional complexities to farming practices can have added financial costs (purchasing multiple pesticides, or disease‐resistant strains, etc.), and may be less effective at controlling outbreaks in the short term. Selecting the most appropriate control strategy for a given system involves weighing these costs against the long‐term gains achieved by delaying the evolution of resistance (Coates, 2023; Gould et al., 2018).

Although individual farms are often managed as independent units, pest population dynamics can span across multiple farm sites. Thus, the risk of resistance evolving on any one farm will depend on pest immigration from nearby farms, and the evolutionary processes occurring at those farms (Rimbaud et al., 2018). The benefits of integrated pest management at one location will be undermined by the selection for and transmission of resistant strains from elsewhere. In these situations, efforts to prevent pest resistance need to encompass large regions containing a network of many farm sites. In principle, integrated pest management can be achieved over a large scale by coordinating treatments across multiple farm sites. In practice, such coordination becomes a complex process, and making decisions at this level requires the ability to predict large‐scale dynamics of the pest in response to different management scenarios (Kragesteen et al., 2019).

A better understanding of the patterns and drivers of pest adaptation is particularly important for the aquaculture sector. Research into emerging aquatic diseases is lagging behind the rapid growth in the scale and diversity of aquaculture (Bouwmeester et al., 2021; Krkošek, 2017). Many aquaculture systems are characterised by the free movement of water between farms and the external environment, permitting transmission of infectious diseases over long distances (Krkošek, 2017). This movement of water facilitates parasite gene flow between farms, allowing adaptive genotypes to disperse through entire aquaculture regions (Besnier et al., 2014; Fjørtoft et al., 2021). In these systems, integrated pest management needs to be considered across multiple farms at the metapopulation level, rather than the individual farm level (Coates, 2023).

Evolutionary models offer the opportunity to explore how the pressures experienced on farms shape the epidemiology and evolution of parasites across very large spatial and temporal scales; scales that would be difficult to test empirically (Haridas & Tenhumberg, 2018; Liang et al., 2013; Onstad et al., 2013; Sisterson et al., 2005). Coates et al. (2022) constructed a model that simulated the metapopulation dynamics of salmon lice (*Lepeophtheirus salmonis*) infesting Atlantic salmon (*Salmo salar*) farms in southern Norway. Salmon lice are the most significant parasite affecting salmonid aquaculture, posing major economic, ecological and welfare problems (Torrissen et al., 2013). Lice have repeatedly evolved resistance to chemical pesticides, and so a diverse array of technologies are deployed to keep outbreaks under control (Barrett, Oppedal, et al., 2020; Coates, Phillips, et al., 2021). Lice can travel for weeks on ocean currents in their planktonic larval phase, allowing infestations to be transmitted between farms that are many kilometres apart (Asplin et al., 2014; Johnsen et al., 2014). The Norwegian coast is divided into 13 ‘production zones’ (Samsing et al., 2019). According to government regulations, farms within a zone are only permitted to increase their salmon production if the estimated impact of the zone on wild salmonid populations – through louse infestation pressure – is low (Myksvoll et al., 2020). Likewise, high‐louse pressure on wild salmonids means that farms within a zone are required to decrease production. Effective louse control within each zone is therefore a priority for the industry.

Coates et al. (2022) parameterised their model using real‐world data to simulate a specific case study: the rapid evolution of resistance to the pesticide azamethiphos, as observed in Norway during the 2000s (Kaur et al., 2017). While the previous article showed that the model could recapitulate past patterns, in this study we investigate a range of alternative management scenarios, to better predict the future eco‐evolutionary dynamics of this parasite. Here, we model a variety of louse prevention and control methods and compare their effects on louse evolutionary dynamics. We examine both ‘discrete’ and ‘continuous’ strategies. A discrete strategy immediately removes lice from salmon during a one‐off delousing event – this includes pesticide baths, mechanical delousing and thermal delousing (Coates, Phillips, et al., 2021). By contrast, continuous strategies act on lice over an extended period. These include structural changes to the sea cage that reduce the chance of lice attaching to a host to begin with (Barrett, Oppedal, et al., 2020). Continuous strategies also include changes to the host itself that reduce louse attachment, or survival shortly after attachment, through the use of functional feeds, selective breeding of louse‐resistant salmon, and technologies in development such as gene‐edited salmon and vaccines (Barrett, Oppedal, et al., 2020; Robinson et al., 2023). Control strategies differ in how they target lice in their life cycle. Delousing treatments vary in which louse life stages are removed and which are left unaffected (Coates, Phillips, et al., 2021). Strategies that alter the host biome or expose lice to certain environmental conditions (e.g., ultra‐violet light) can impose sublethal effects such as delayed development or reduced fecundity (Barrett et al., 2019; Covello et al., 2012; Grayson et al., 1995; Robinson et al., 2023). In this study, we adjust parameters that determine the type of management strategy (e.g., whether it is deployed discretely or continuously through time; how the treatment targets the louse life cycle) to observe their effects on louse epidemiology and evolution.

Furthermore, we adjust parameters associated with the louse allele for resistance, to assess how these parameters drive metapopulation and evolutionary dynamics. These include the extent of the advantage conferred by the resistant allele (which in turn determines the strength of selection by treatments), the dominance of the allele (whether there is a dominant, recessive or additive effect), and the frequency of the allele in the metapopulation at the beginning of the simulation (including the location the mutation for resistance initially arises). Resistance is expected to rapidly evolve with some combinations of these factors and to be lost through genetic drift with other combinations. We aim to identify factors which have the greatest influence on accelerating or suppressing adaptation. Our findings will contribute to the broader knowledge of louse evolutionary dynamics, with which the industry and regulators can design management strategies that minimise the opportunity for widespread resistance to evolve.

## 2 METHODS

We built upon the metapopulation evolution model described in Coates et al. (2022).

**FIGURE 1:**
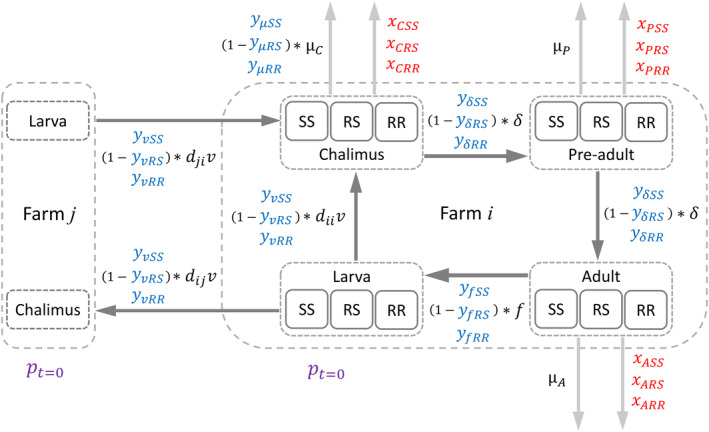
A simplified diagram of the metapopulation model, showing the partitioning of lice by genotype (SS, RS, SS; where S is the susceptible allele and R is the resistant allele), life stage (larva, chalimus, pre‐adult, adult) and farm. Lice transition between life stages (dark arrows) and are lost through mortality (light arrows) each time‐step. Transitions are mediated by parameters for: the dispersal probability of larvae between farms, *d*, the host attachment success of larvae, *v*, the loss of lice through background mortality, *μ*, the weekly rate of development to the next life stage, *δ*, and the weekly reproductive output of adults, *f*. Continuous strategies reduce the above parameters by a corresponding efficacy parameter, *y* (given in blue). Parameters *y* are assigned independently for each of the three genotypes, to explore different selection differentials. Discrete management strategies induce treatment mortality, *x*, for each life stage and genotype (given in red). The gene frequency of the R allele at the beginning of the simulation, *p*_*t*=0_, was adjusted in simulations (by assigning the starting proportion of heterozygotes). Depending on the simulation, *p*_*t*=0_ was either constant across all farms or differed between farms.

This model is an individual‐based, stage‐structured matrix model that tracks louse numbers over discrete, weekly time steps, *t*. Lice are grouped according to genotype (*g*), life stage (*b*) and farm (*i*; Figure 1). Our metapopulation was comprised of 537 populations, representing farm sites throughout southern Norway (58.4–66.4° N). The transmission of free‐living larvae between farms was parameterised using particle‐tracking model outputs produced for this region by Samsing et al. (2017). This particle‐tracking model, developed by the Norwegian Institute of Marine Research, combines advanced hydrodynamic processes and biological data and has been widely used to simulate the dispersal of lice on currents along the Norwegian coastline (Johnsen et al., 2021; Myksvoll et al., 2018; Sandvik et al., 2020).

Lice were grouped into ‘larva’ (representing the free‐living stages), ‘chalimus’, ‘pre‐adult’ and ‘adult’ life stages, notated here as $b=\left\{L,C,P,A\right\},$ respectively. At each time step, a proportion of lice, *δ*, progressed to the subsequent life stage (Figure 1). The value of *δ* was calculated according to the temperature, *T* (°C), at the corresponding farm and time‐step. We used the equation given by Hamre et al. (2019) to calculate the daily transition rate of lice (constants averaged across sexes), which was then converted into a weekly rate (Table 1).

**TABLE 1:** Summary of model parameters.

Indices	Description	Value
*t*	Time‐step	Represents 1 week.
*b*	Life stage	Lice categorised as Larva, Chalimus, Pre‐adult or Adult: $b\in \left\{L,C,P,A\right\}$
*g*	Genotype	Lice categorised as SS, RS or RR: $g\in \left\{\mathrm{SS},\mathrm{RS},\mathrm{RR}\right\}$
*i*	Farm	Lice categorised by farm location (with 537 farms in metapopulation): $i\in \left\{1,2\ldots 537\right\}$
Life cycle parameters		
*T*_*ti*_	Temperature (°C) at farm *i* and time‐step *t*	Average temperature over five‐week periods Data from barentswatch.no (see Appendix S1)
*δ*_*T*_	Proportion of lice developing to next life stage per week	Calculated according to temperature, *T*_*ti*_ Daily transition rate (from Hamre et al., 2019): $\delta_{\mathrm{day}}=0.000581T^{2}+0.0094805T+0.0047395$ Weekly transition rate: $\delta =1-{\left(1-\delta_{\mathrm{day}}\right)}^{7}$
*f*_*T*_	Number of larvae produced per adult per week	Calculated according to temperature *T*_*ti*_ Number of eggs per egg‐string (from Johnsen et al., 2020): $N_{\text{eggs}}=e^{\left\{5.6-0.43*\frac{T}{10}-0.78*{\left[\ln\left(\frac{T}{10}\right)\right]}^{2}\right\}}$ Days between clutches (from Johnsen et al., 2020): $D_{\text{hatch}}=0.25*\left[\frac{5}{4.85e^{-4}*T^{2}+8.667e^{-3}*T+3.75e^{-3}}\right]$ Number of larvae per adult per week: $f=\left(N_{\text{eggs}}/D_{\text{hatc}h}\right)*7$
*μ*_*b*_	The proportion of life stage *b* lost per week through background mortality	Weekly mortality: $\mu_{C}=0.014$ $\mu_{P}=0.162$ $\mu_{A}=0.162$ Estimated from daily mortality data (Stien et al., 2005). See Appendix S1
*d*_*ji*_	The proportion of larvae dispersing from farm *j* to farm *i*	Values taken from particle‐tracking model outputs (Samsing et al., 2017) for each unique combination of *j* and *i*
*v*	The proportion of incoming larvae that attach to host	v=0.05 See Appendix S1
Treatment parameters		
*x*_*bg*_	The proportion of stage *b* and genotype *g* removed by discrete strategy	Values are adjusted across simulations, according to treatment efficacy and selection differential.
*y*_*vg*_	Proportion reduction in larval attachment (*v*) of genotype *g* by a continuous strategy	
*y*_*δg*_	Proportion reduction in development rate (*δ*) of genotype *g* by a continuous strategy	
*y*_*μg*_	Proportion reduction in background mortality survival (1 − *μ*_*C*_) of genotype *g* by a continuous strategy	
*y*_*fg*_	Proportion reduction in reproductive output (*f*) of genotype *g* by a continuous strategy	

A proportion of lice, *μ*, was also lost each week through a background mortality rate (Figure 1). We used the lower range of daily mortality rates estimated by Stien et al. (2005), averaged across sexes. These were converted into weekly background mortality rates for chalimi, pre‐adults and adults (Table 1).

The parameter *f* is the mean number of larvae produced per adult per week. The value for *f* was calculated at each farm and time step according to the corresponding temperature, using the equations given in Johnsen et al. (2020) (Table 1).

The larvae produced at *t* are subsequently dispersed across farms at *t + 1*. The proportion of larvae transmitted from any given farm *j* to any given farm *i* is captured by the parameter *d*_*ji*_ (Figure 1). The dispersal probabilities for each farm pair were provided by particle‐tracking model outputs (Samsing et al., 2017). Of the copepodids that disperse to a farm site, a proportion, *v*, successfully re‐enters a cage and attaches to a host (Table 1).

Lice were grouped according to genotype, *g*, at a single, biallelic locus. Genotypes are notated here as $g=\left\{\mathrm{RR},\mathrm{RS},\mathrm{SS}\right\}$, where R is the resistant (mutant) allele and S the susceptible (wild type) allele. We focus on single‐locus adaptive traits in lice with Mendelian inheritance. Single‐locus mutations are responsible for pesticide resistance in many pest species, including organophosphate resistance in salmon lice (Groeters & Tabashnik, 2000; Kaur et al., 2015). Resistance to other treatments may be polygenic (Haridas & Tenhumberg, 2018; Holt & Hochberg, 1997; Stear et al., 2001). Our focus on single‐locus traits nevertheless provides a groundwork for understanding how different biotic and abiotic factors drive louse adaptation across a farm network. There is non‐assortative mating of adults, and the offspring (larval) genotype frequencies are the Hardy–Weinberg proportions.

In some scenarios, farms used a discrete management strategy to remove lice. In these simulations, delousing occurred when the adult louse abundance on a farm exceeded one adult louse fish^−1^, or 0.4 adult louse fish^−1^ during weeks 16–21 of the year. These correspond to Norway's legal lice limits of 0.5 adult females fish^−1^, or 0.2 adult females fish^−1^ in spring (Sandvik et al., 2021), assuming a 1:1 sex ratio in adults. The proportion of louse mortality from a discrete treatment (i.e. the treatment efficacy) was given by the parameter *x*, which could be assigned unique values for each life stage and genotype (Figure 1). As in Coates et al. (2022) we also included a ‘forced harvest’ of farms (resulting in 100% mortality of all attached lice) when abundance exceeded 2 adults fish^−1^.

A new aspect of the model was the capacity to assign farms a continuously‐acting management strategy with various lethal or sublethal effects. In these scenarios, the model parameters *v*, *μ*_*C*_, *δ*, or *f* could be adjusted by *y*_*v*_, *y*_*μ*_, *y*_*δ*_, or *y*_*f*_, respectively (Figure 1). Different *y* values could be assigned for each genotype. The values for *y* are the proportional reductions (i.e., treatment efficacy) in the related parameter. For example, if a continuous strategy reduced the fecundity of SS adults by 75% (*y*_*f*SS_ = 0.75), the reproductive output of SS adults on that farm would be (1–0.75)*f. Continuous strategies affect a farm population every time step and are not influenced by mandated lice limits.

Further details on the underlying structure and functions of the matrix model can be found in the Appendix S1.

### 2.1 Simulations

We ran simulations over time steps equivalent to 15 years. The number of lice present at the start of the simulations was determined by the average louse abundance for each farm in the first week of the year (from the barentswatch.no database; Coates et al., 2022).

In the outputs of each simulation, we were particularly interested in (1) mean adult louse abundance, (2) delousing frequency, and (3) the gene frequency of the resistant R allele, as metrics of the louse population. First, adult abundance indicates average infestation pressure across farms, as well as the overall size of the louse metapopulation, since the number of hosts was kept constant through time for each farm. Second, delousing frequency measures the severity of outbreaks, since farms are treated when lice limits are exceeded. There are financial costs to using any delousing technology, including the loss of stock through salmon mortality (Overton et al., 2019). Simulations with frequent treatments therefore indicate expensive, inefficient, and unsustainable louse management. Third, the frequency of the R allele in the metapopulation tracks the rate at which lice adapt to a strategy. In addition to looking at these metrics at the metapopulation level, we also compared outputs for different salmon production zones. Our study area contains a total of 537 farm sites, covering 8 of the 13 Norwegian production zones.

### 2.2 Type of management strategy (without selection)

First, we ran simulations without any genetic or phenotypic variation in the louse population, to compare different types of louse management. In these simulations, all farms used either (1) a single type of discrete delousing treatment, or (2) a continuous management strategy *plus* a discrete treatment. Discrete treatments were only applied when adult louse abundance exceeded the farm limit.

In the first set of scenarios, delousing treatments differed in which louse life stages they removed, and in their efficacy at removing those stages. The treatment targeted either all parasitic stages (as with pyrethroids, mechanical delousing and emamectin benzoate), only motile stages (as with organophosphates, hydrogen peroxide, and thermal delousing), or only immature (chalimus and pre‐adult) stages (as with chitin synthesis disrupters; Coates, Phillips, et al., 2021). The efficacy of the treatment against the target stages ranged in the simulations from 10% to 99% (*x*_*b*_ = 0.1 − 0.99).

In the second set of scenarios, we tested how the addition of a continuous management strategy (when there was already a discrete strategy in use) affected the metapopulation. We compared simulations in which the continuous management strategy reduced either (a) the success of copepodid attachment, (b) chalimus survival, (c) louse development rate, or (d) adult reproductive output. For this, we included either *y*_*v*_, *y*_*μ*_, *y*_*δ*_, or *y*_*f*_ (Figure 1) into the model. The efficacy of the strategies against the target parameter ranged in the simulations from 25% to 75% (*y* = 0.25–0.75). The additional discrete strategy was kept the same in these scenarios: it removed 90% of adult and pre‐adult lice, and 75% of chalimi. We chose these values to approximate the effect of mechanical delousing (Flatsetsund Engineering AS, 2017; Gismervik et al., 2017).

### 2.3 Selection gradients, the starting frequency of resistance and dominance effects

In the next set of simulations, we included genetic variation in the susceptibility of lice to a management strategy. We included three louse genotypes at a single, biallelic locus: the wild‐type SS, the heterozygote RS, and the resistant RR genotypes.

In one group of simulations, all farms only used a discrete delousing strategy, which removed a proportion of lice from all parasitic stages. Lice with the R allele had higher proportional survival to the treatment. In a second group of simulations, all farms used a continuous management strategy, which reduced the attachment success of copepodids. Lice with the R allele had higher attachment success. Farms using the continuous strategy also deployed mechanical delousing (with 90% and 75% efficacy against motile and chalimus stages, respectively) whenever lice limits were exceeded, but here mechanical treatment did not impose any selection on lice. In a third group, all farms used a continuous management strategy as above, but this was not supplemented by any discrete delousing (although there was still a forced harvest of the farm at 2 adults fish^−1^; Coates et al., 2022).

In these scenarios, we assessed how the evolution of resistance was influenced by the type of strategy (discrete vs. continuous; the life stages targeted), the efficacy of the strategy, the relative fitness of the R allele, and any interaction between these factors. The proportion of treatment survival/attachment of SS lice, (1 − *x*_SS_) or (1 − *y*_SS_), ranged in the simulations from 0.3 to 0.6. We compared two relative fitness gradients. The survival/attachment of RR lice was maintained in each scenario at either 1.5 or 3 times that of SS lice. In all scenarios, the R allele had an additive effect, so that the fitness of heterozygotes was midway between that of the homozygotes. As in Coates et al. (2022), the resistant allele, R, started at a very low frequency (*p* = 0.005) throughout the metapopulation.

After this, we examined more deeply the influence that the selection strength has on the time taken for the frequency of the R allele to reach 90% in the adult metapopulation. These simulations explored the effect of (1) treatment type, (2) the initial frequency of resistance, and (3) the dominance of the R allele. In these, all farms used a continuous strategy that reduced either copepodid attachment, fecundity, weekly chalimus survival, or the development rate of SS lice by 75%. The relative fitness of RR lice under the strategy ranged from one time (i.e., no advantage of the R allele) to four times that of SS lice. Relative fitness of four, for example, meant that the attachment, survival, development rate, or fecundity (whichever is under selection) of RR lice was four times that of SS lice.

The starting frequency of the R allele ranged in simulations from *p* = 0.005 to *p* = 0.1. We adjusted *p* by changing the proportion of heterozygotes present at the start of the simulation. This approach was informed by pilot simulations in which the starting ratio of RR to RS genotypes was varied (ranging from only homozygotes to only heterozygotes), whilst keeping the frequency of the R allele constant. The results of these simulations showed that metapopulation dynamics (such as the rate of evolution, louse abundance levels and treatment frequency) were the same regardless of genotype ratios, as long as the overall R frequency was the same. This was the case for additive, dominant and recessive resistance scenarios.

We also compared the additive, dominant and recessive effects of the R allele. For these simulations, we focused on the continuous strategy that reduced copepodid attachment. We repeated the above simulations but with a complete dominant or recessive effect of the resistant allele. When there was a dominant effect, heterozygotes had the same attachment success as RR lice. When recessive, heterozygotes had the same success as SS lice.

### 2.4 Spatial heterogeneity in the starting frequency of resistance

To explore the spatial dynamics of evolution more deeply, we then ran scenarios in which the R allele was initially found in only a small subset of the study area. In each simulation, there was a 0.5° band in latitude in which the R allele occurred at low frequency (1% of lice on farms within this band had the RS genotype, equivalent to *p* = 0.005). The remaining farms initially contained only SS lice. In simulations looking at resistance to a discrete strategy, treatments were 90% effective against SS lice, and 20% for RR lice (across all parasitic stages). We compared simulations in which the R allele had either an additive, dominant or recessive effect. In simulations looking at resistance to a continuous strategy, there was 50% efficacy against SS copepodid attachment, but no effect for RR copepodids. We assigned strong selection gradients in these scenarios so we could observe any evolutionary patterns clearly over the 15‐year simulation period.

## 3 RESULTS

### 3.1 Type of management strategy (without selection)

There was a seasonal cycle in infestation levels: the louse metapopulation grew over summer, and shrunk over winter and spring. After 1 year in each of the simulations, the seasonal cycle in metapopulation size remained consistent between years. Likewise, the number of delousing treatments applied to farms each year was also constant after the first year. The equilibrium reached by the metapopulation was affected by the type and efficacy of the louse treatment.

**FIGURE 2:**
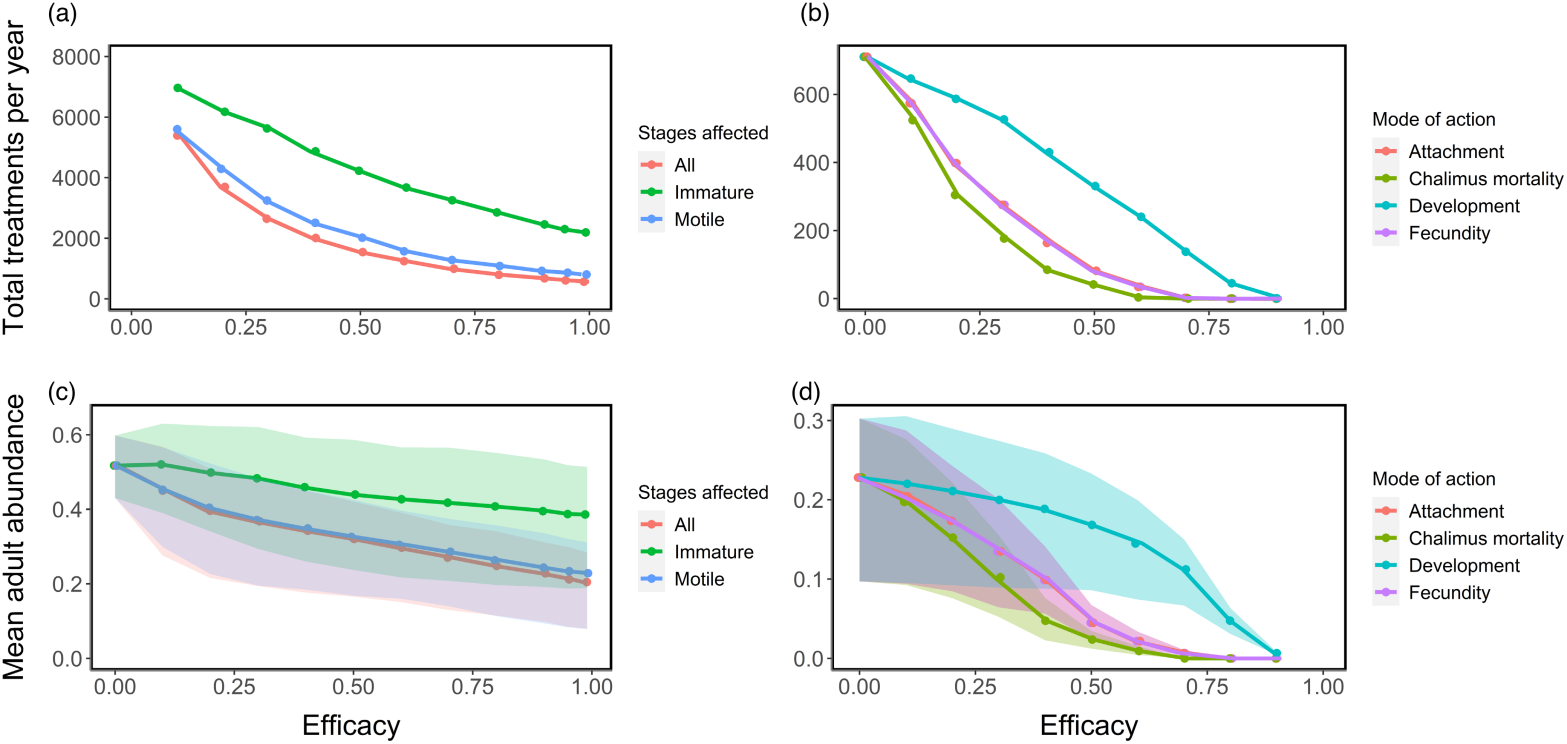
The total number of delousing treatments applied across all farms per year (a, b), and the mean adult abundance (lice per fish) per year (c, d), with different efficacies of a louse control strategy. The control was either a discrete strategy that targeted different louse life stages (a and c), or a continuous strategy with different modes of action on the louse life cycle (b and d). The continuous strategy was supplemented by mechanical delousing with a fixed efficacy (see Methods). Shading represents the upper and lower mean abundance over one seasonal cycle. These simulations assume no evolution of resistance to either discrete or continuous strategies.

Increasing the efficacy of discrete delousing reduced the total number of treatments required and the average adult infestation level throughout the seasonal cycle (Figure 2). For example, increasing the efficacy of a treatment (targeting all life stages) from 10% to 99% reduced yearly treatments by 90% (from approx. 5400 to 570 per year), and mean abundance by 50% (from approx. 0.45 to 0.23 adults fish^−1^). Discrete treatments that removed only the motile life stages were only slightly less effective at louse control than those that removed all stages on a farm. By contrast, treatments that only removed immature stages were much less effective; their efficacy against immature lice needed to be much greater to reduce infestations to a similar level as the other treatments (Figure 2a,c).

The effectiveness of a continuous management strategy at controlling lice at a metapopulation level also varied depending on how it acted on the parasite. Reducing weekly chalimus survival was most successful at driving down adult abundance and, in turn, the frequency of mechanical treatments in response to high abundances (Figure 2b,d). Strategies that either reduced copepodid attachment or reduced fecundity had the same outcome on the metapopulation. Slowing the louse development rate had the smallest impact.

For example, without any continuous strategies, approximately 700 delousing treatments (equivalent to mechanical delousing) were used per year. To limit the number of delousing treatments to approximately 100 per year, an additional continuous strategy would need an effect size of approximately 40% on chalimus survival, 50% on attachment or fecundity, or 75% on development rate.

With a ≥70% reduction in weekly chalimus survival, or with a ≥80% reduction in copepodid attachment or fecundity, lice were eradicated from the study system after 3–11 years.

**FIGURE 3:**
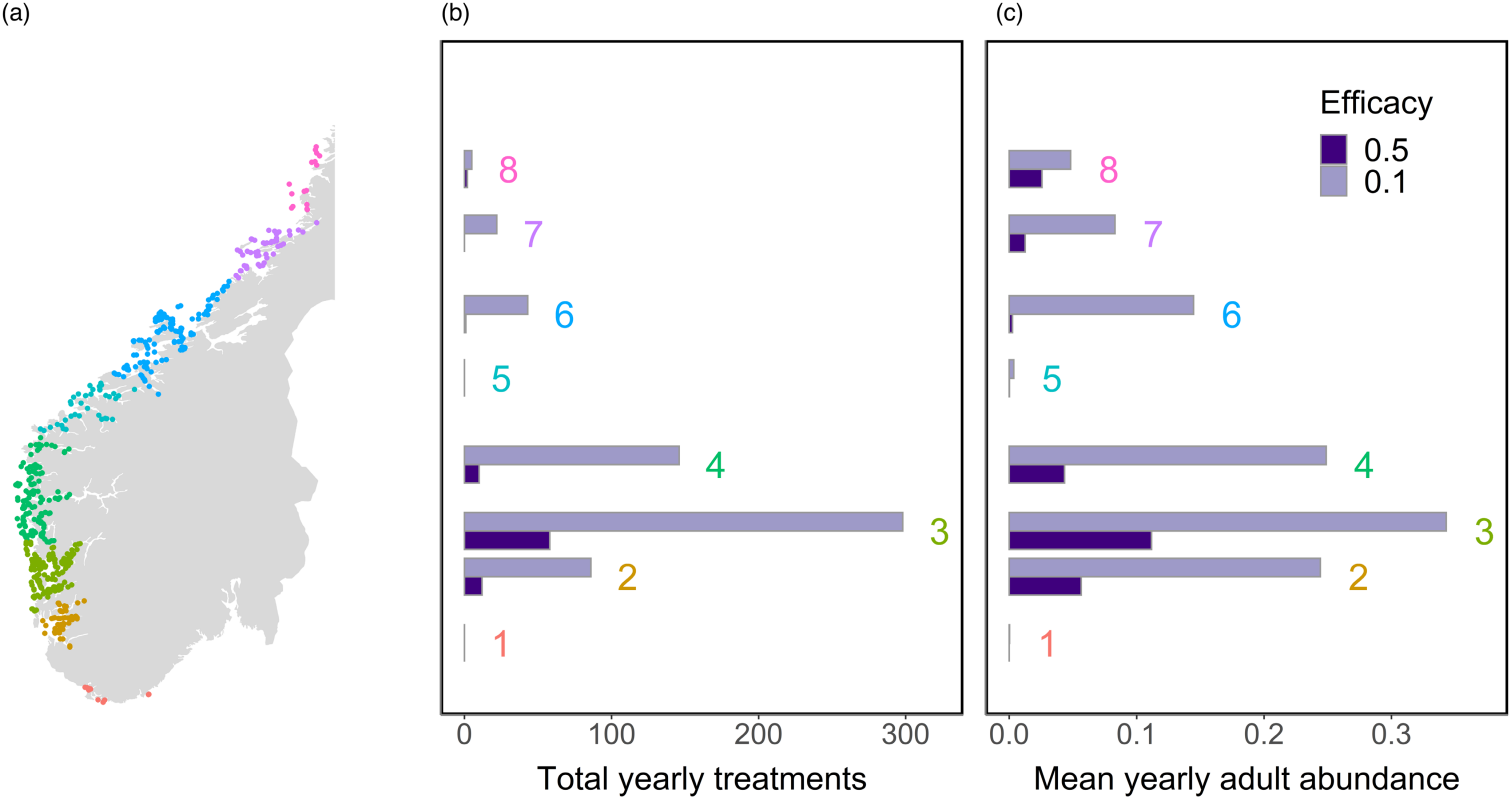
(a) Location of Production Zones 1–8 in Norway (colours). A yearly number of discrete delousing treatments (b) and yearly mean adult abundance (c) by production zone, under a continuous strategy with either 50% or 10% efficacy against larval attachment.

In all simulations, the highest infestations and most frequent delousing treatments occurred in Production Zone 3 in the south‐west of Norway, followed by the adjacent Zones 2 and 4 (Figure 3). Increasing the efficacy of the management strategy reduced infestations across all Production Zones. Infestations were kept low in Zones 1 and 5, even with relatively low‐treatment efficacy.

### 3.2 Strength of selection and the starting frequency of resistance

**FIGURE 4:**
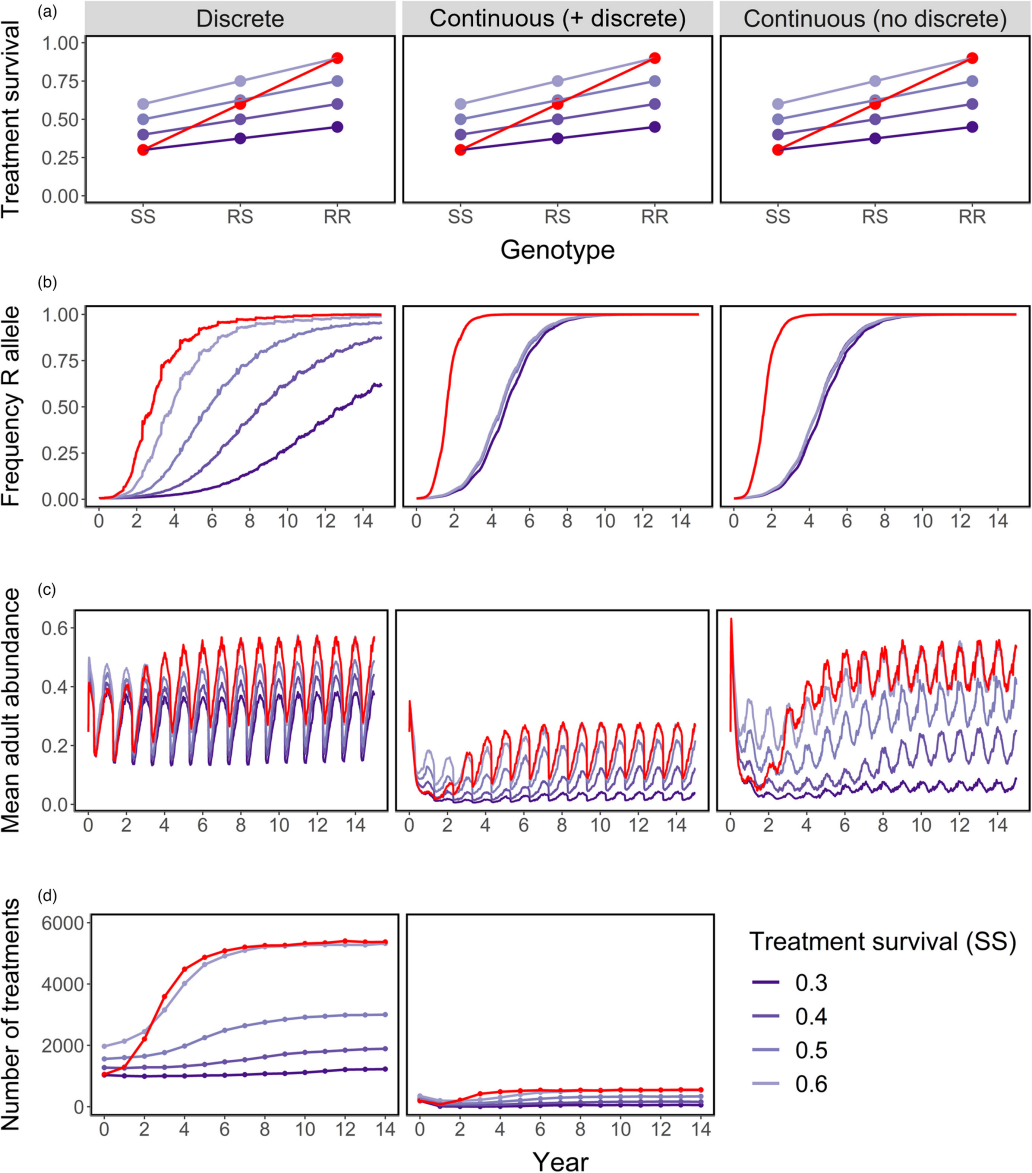
(a) The proportion survival from discrete treatments (or attachment success under continuous treatments) of the three louse genotypes. Each line represents one simulation. Purple lines = the survival of RR lice was 1.5 times that of SS lice. Red line = survival of RR lice was 3 times that of SS lice. (b) Frequency of the R allele in the entire adult metapopulation over 15 years in each scenario. (c) The mean adult abundance (lice per fish) across all farms. (d) The total number of discrete delousing treatments applied to farms each year.

Treatment survival of the RR genotype was either 1.5 or 3 times that of the SS genotype (given by the purple and red lines, respectively, in Figure 4), with the latter corresponding to a stronger selection gradient. The rate at which resistance evolved in the metapopulation (the increase in the frequency of the R allele) was faster with a steeper gradient (Figure 4b). When the R allele was selected by a discrete strategy, the rate of evolution was fastest under the steeper selection gradient, but it was also faster when the efficacy of the treatment was lower. By contrast, with selection imposed by a continuous strategy, the selection gradient alone directly translated to the rate at which the R allele approached fixation. In all simulations, once the frequency of the R allele reached approximately 0.9, the metapopulation reached equilibrium – the yearly cycle in mean abundance and the yearly treatment frequency were constant between the years. The efficacy of the strategy against SS lice determined the extent to which infestations (and hence treatments) were reduced at the beginning of the simulation (Figure 4c,d). The efficacy against RR lice determined the infestation and treatment levels once the RR allele approached fixation.

**FIGURE 5:**
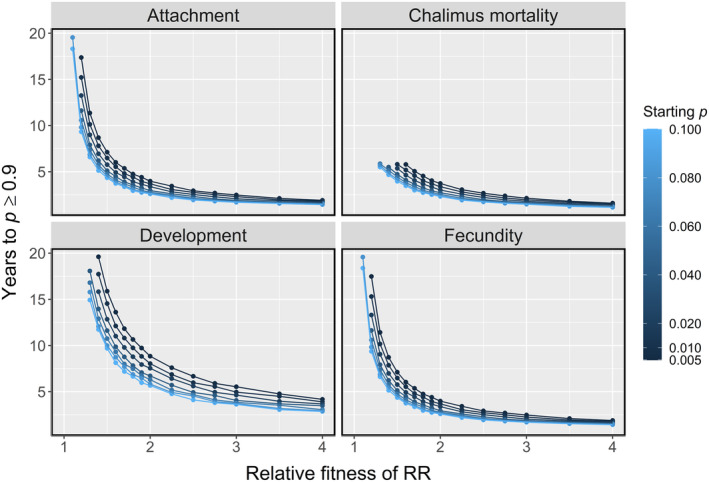
The number of years taken for the frequency of the R allele in the metapopulation (*p*) to reach ≥0.9, given: the relative fitness of the RR genotype (i.e., how many times higher attachment, survival, development or fecundity for RR lice is than for SS lice), the frequency of R (*p*) at the beginning of the simulation, and whether selection was imposed by a strategy reducing either copepodid attachment, chalimus survival, louse development or fecundity.

Under selection by a continuous strategy, as the relative fitness of RR lice was increased from 1.1 to 2 times that of SS lice, the time until *p* > 0.9 was dramatically shortened. Increasing the relative fitness gradient above 2 continued to accelerate evolution, but more slowly (Figure 5). As expected, when *p* started higher at the beginning of the simulation, it took less time for it to reach 0.9. The effect that the initial *p* had on the time until R was fixed was greater in scenarios with weaker selection.

The strategy that reduced the louse development rate showed a slower evolution of resistance than the other strategies. Strategies affecting copepodid attachment, chalimus mortality and fecundity all drove resistance to evolve at the same rate, when under the same relative fitness gradient and starting *p*. The exception was when the fitness of the RR genotype was ≤1.6, relative to the SS genotype (or ≤1.3 at higher starting *p*), during which the strategy affecting chalimus survival eradicated lice from the metapopulation before resistance could evolve. In all other simulations, the R allele was gradually lost from the metapopulation via drift when it conferred no fitness advantage (relative fitness = 1).

**FIGURE 6:**
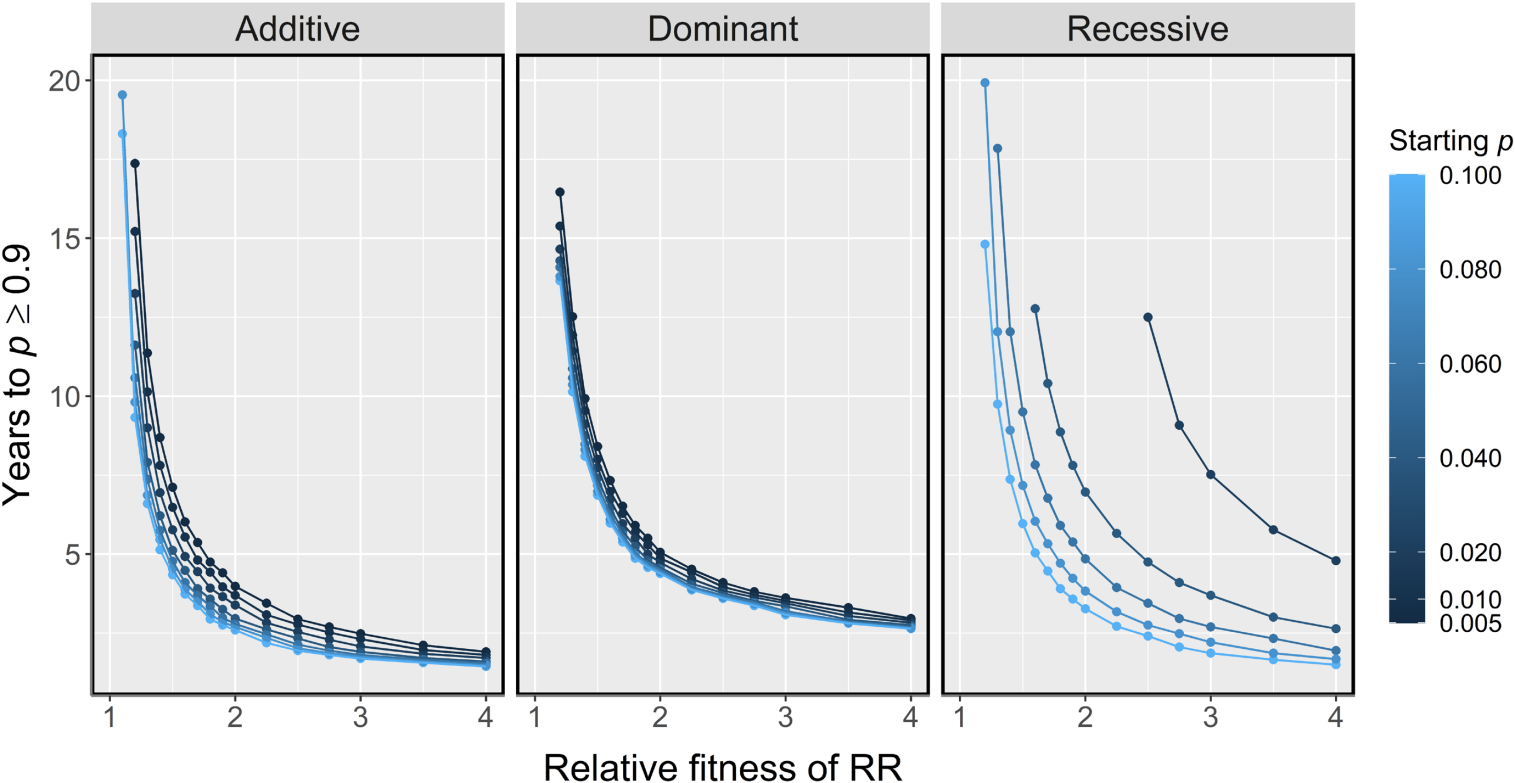
The number of years taken for the frequency of the R allele in the metapopulation (*p*) to reach ≥0.9, given: the relative fitness of the RR genotype (relative to the SS genotype), the frequency of R (*p*) at the beginning of the simulation, and whether the R allele had an additive, dominant or recessive effect. The selection was imposed by a continuous strategy that reduced the attachment of SS lice by 75%.

**FIGURE 7:**
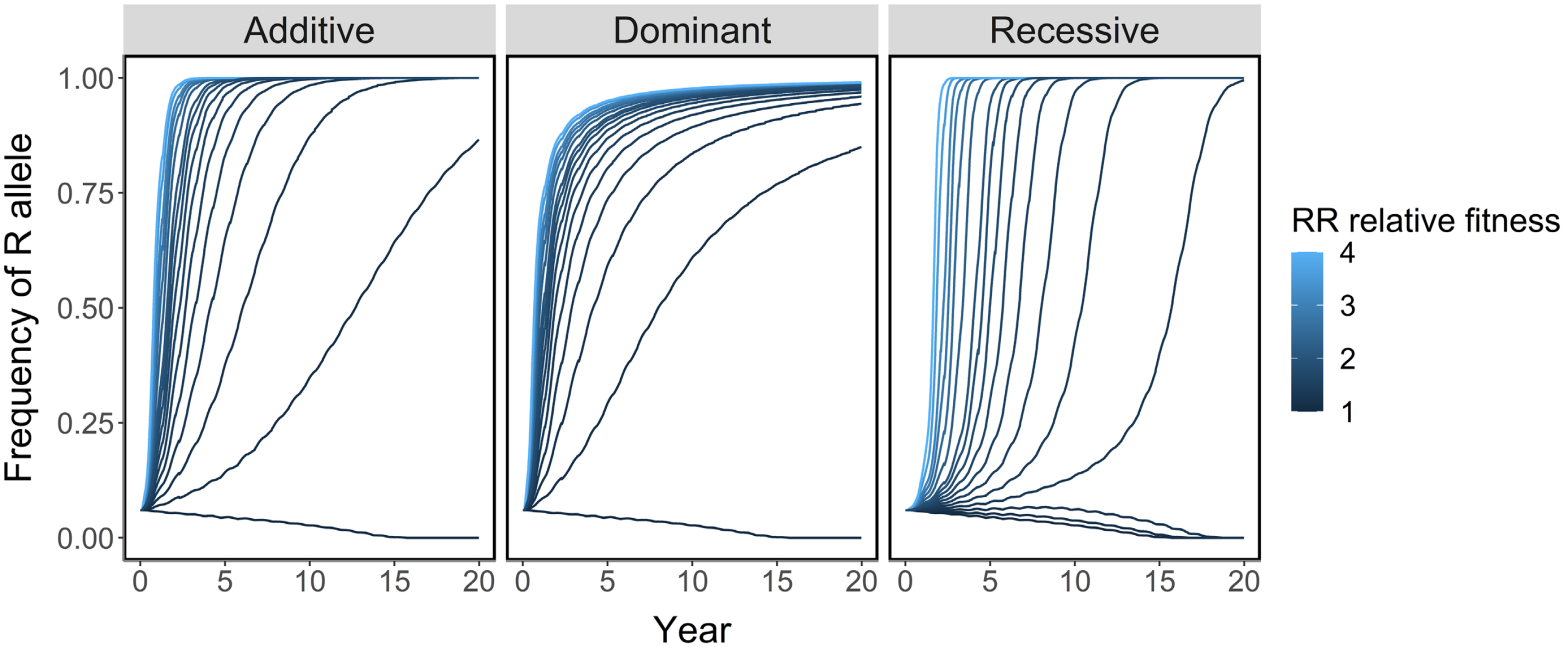
Frequency of R in the metapopulation (*p*) over 20 years with an additive, dominant or recessive effect of the R allele. Colour = relative fitness, with the attachment success of the RR genotype ranging 1–4 times that of SS lice. The selection was imposed by a continuous strategy that reduced the attachment of SS lice by 75%. The initial frequency of the R allele was *p* = 0.06.

Our predicted allele trajectories match those expected for modelling additive, dominant and recessive effects (Dehasque et al., 2020; Teshima & Przeworski, 2006). When resistance was dominant, the frequency of the R allele began to plateau earlier than with an additive effect, so it took longer for *p* to reach 0.9 (Figures 6 and 7). When resistance was recessive, the increase in *p* started slowly but accelerated towards rapid fixation of R. The initial frequency of R was more important in the rate of evolution when resistance was recessive. At a higher starting gene frequency (*p* ≥ 0.08), recessive resistance evolved at a similar rate to dominant resistance (Figures 6 and 7). At a lower gene frequency, it took much longer before the R allele spread through the metapopulation. The recessive R allele was lost from the metapopulation altogether via drift under scenarios with very low relative fitness and/or starting frequency (Figure 7).

### 3.3 Spatial heterogeneity in the starting frequency of resistance

**FIGURE 8:**
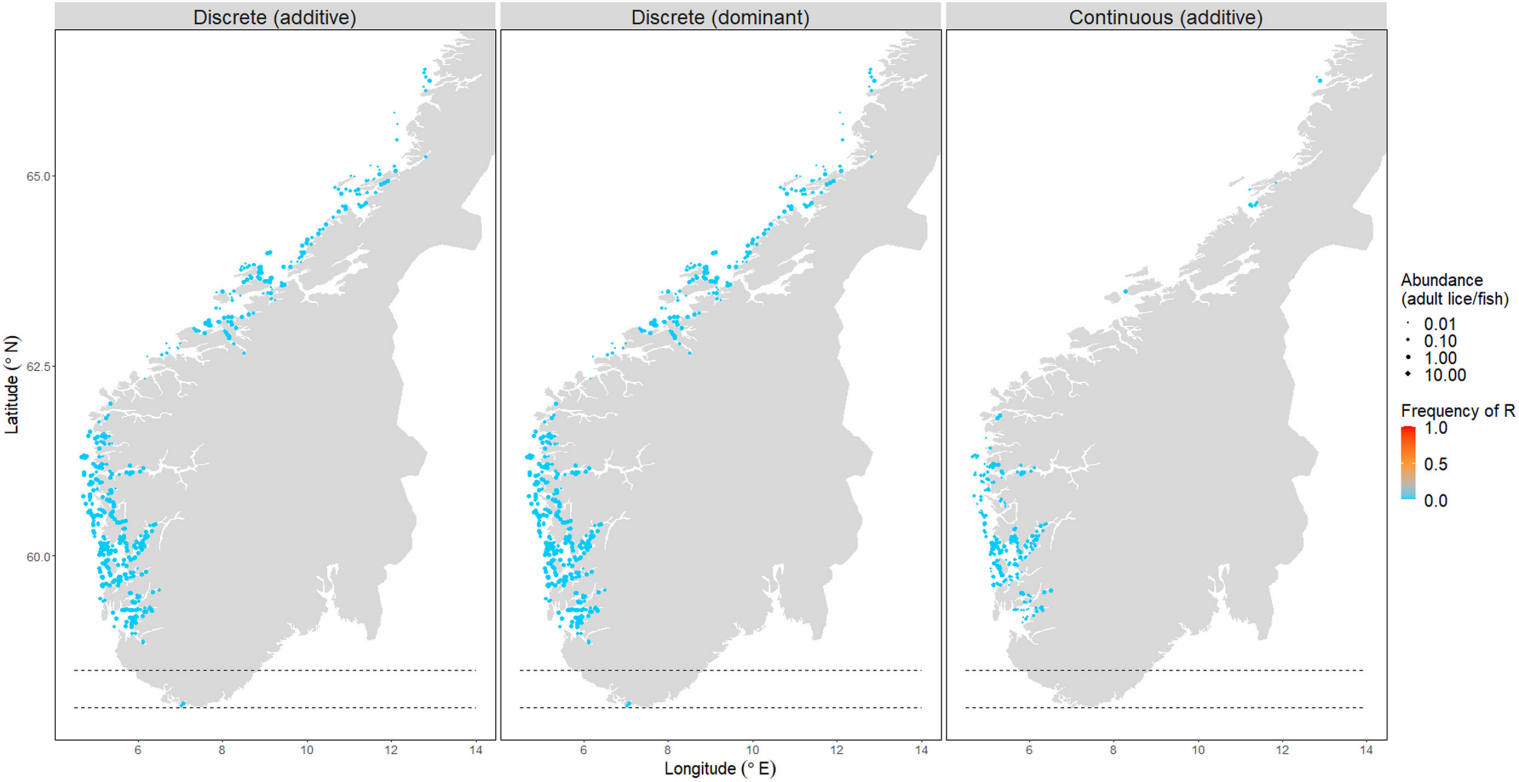
Animation is available in Figure S1. The adult salmon louse metapopulation on Norwegian farms after 15 years of selection. The colour indicates the frequency of the R allele on a farm (blue = susceptible, red = resistant population). The size of points represents adult abundance. Each frame represents the results from one simulation, in which the R allele was initially found only on farms within the 0.5° latitude band indicated by the dashed lines. Panels for (a) additive resistance to a discrete strategy, (b) dominant resistance to a discrete strategy, (c) additive resistance to a continuous strategy.

**FIGURE 9:**
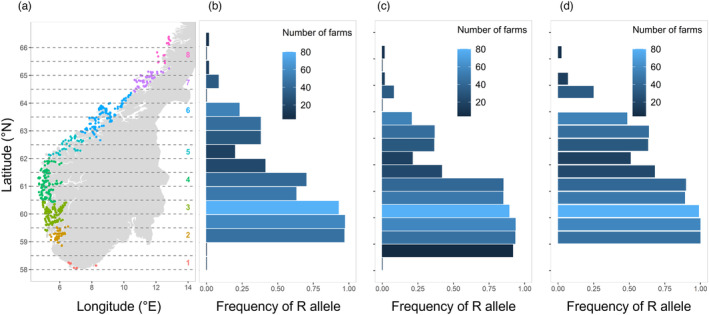
(a) Locations of farm sites in the study area (colours = Production Zones 1–8), and the 0.5° latitude bands (between dashed lines) in which the R allele initially occurred in different simulations. The frequency of the R allele (in the entire adult metapopulation) after 15 years of selection by a management strategy, when the R allele was initially found in each of these bands. The colour indicates the number of farm sites within each band (that started with heterozygous lice). (b) Additive resistance to a discrete strategy; (c) dominant resistance to a discrete strategy; (d) additive resistance to a continuous strategy.

In the region where the R allele originated, resistant homozygotes became the most common genotype within five years in most simulations. The exceptions were simulations with resistance starting in bands within Production Zone 1 (~58.25° N), Production Zone 5 (~62.5° N) or the north of Production Zone 6 (64.25° N), in which louse populations retained their susceptibility (Figure 8). In most simulations, resistance rapidly spread northwards to new farms outside of its original location (Figure 8). When the R allele originated in Production Zone 2 in the south, it took 4 years for the R allele to reach Production Zone 8 in the north (>800 km away), and 10 years for the gene frequency of R in the entire metapopulation to increase from *p* = 3.5e^−4^ to *p* = 0.9 (Figure 9).

Despite the strong selection and rapid localised adaptation, resistance tended not to disperse southwards. Lice on farms further south of the region starting with R retained high levels of treatment susceptibility. The exception was when resistance originated in areas within Production Zones 3 and 4, in which case resistance travelled southwards but did not reach as far as Production Zone 2.

Resistance spread through the metapopulation to a similar extent when selected for by a discrete or a continuous strategy (Figures 8 and 9), although it was slightly more rapid under a continuous strategy (under the parameters assigned in these simulations), in part due to the continuous scenario reducing the overall size of the metapopulation more effectively. Resistance dispersed more rapidly and extensively through the metapopulation when the R allele had a dominant, rather than additive, effect. When resistance was recessive, the R allele was lost from the metapopulation, regardless of its initial starting location.

## 4 DISCUSSION

We have used a numerical model to better understand the evolutionary dynamics of salmon lice across a metapopulation of salmon aquaculture sites, under a range of management strategies. By predicting how lice will respond to specific treatment scenarios, our model is an important step towards identifying management approaches that remain durable overtime against the evolution of resistance.

### 4.1 Discrete versus continuous management strategies

We first quantified the effect of different control strategies on the metapopulation dynamics of the parasite. Unlike discrete delousing strategies, which were deployed sporadically in the model through space and time, continuous management strategies imposed a constant pressure on the metapopulation with each time step. When coupled with mechanical delousing (which was used in the model when adult louse abundance exceeded the legal limit), continuous strategies were highly effective at reducing infestations, even with a relatively low efficacy. For example, a continuous strategy is only needed to reduce copepodid attachment or fecundity by 40% in our simulations to halve the entire adult metapopulation. Naturally, as treatment events become more frequent, the suppression of the pest population intensifies – but at the risk of also imposing greater selection (Liang et al., 2013; McEwan et al., 2016).

### 4.2 Effect of strategy on louse life cycle

Discrete management strategies had the greatest success when they removed adults, compared with targeting other life stages. Lice transition through a number of immature stages over a relatively short period before spending many months as adults (Mustafa et al., 2000). Without removal of the final stage, farms accumulate high‐adult loads (culminating in a forced harvest at 2 adults fish^−1^) if more adults are gained through maturing pre‐adults than are lost through background adult mortality. The ineffectiveness of only removing immature lice was amplified by the treatment regime assigned in the model: in our simulations, farms were only treated once adult abundance exceeded a set limit. A treatment that does not remove adults is more practical when used during the early stages of infestation *before* adult abundance reaches critical levels (Branson et al., 2000).

Continuous strategies that targeted either copepodid attachment or reproductive output had the same outcome at the metapopulation level. Both methods remove a proportion of larvae from the system – it did not make a difference whether this occurred at the beginning of the larval stage (with fewer eggs) or at the end (with fewer copepodids attaching to a host). These two strategies had a one‐off effect on a cohort of lice. By contrast, strategies that reduced weekly chalimus survival could remove lice from a single cohort over multiple time steps, since lice spent more than one week in this stage under colder conditions. It is for this reason that a reduction in the parameter for chalimus survival had a greater effect on the metapopulation than the same per cent reduction in the other parameters.

The strategy that slowed the development rate also acted on a cohort of lice over multiple time steps, but its effect on the metapopulation was lower since it did not directly remove lice from the system. Slowing louse development delayed infestations from exceeding farm lice limits, meaning fewer mechanical treatments were needed each year. It also meant more lice were lost through weekly background mortality before they could reach maturity, hence, the reduction in mean adult abundance.

### 4.3 Strength of selection

As expected from previous evolution models, adaptation accelerated as the selection differential increased (Falconer & Mackay, 1996; Lande & Arnold, 1983). With a constant selection pressure – i.e., selection imposed by a continuously acting strategy used across all farms – the rate at which resistance evolved in the population was determined by the relative fitness gradient. By contrast, the speed of adaptation to a discrete strategy was dependent on the absolute fitness values (i.e., treatment efficacy) for each genotype, as well as the relative fitness of genotypes. When the efficacy of a strategy is lower, farms need to be treated more regularly to keep infestations under control (Kragesteen et al., 2023). This results in more frequent selection, which accelerates the evolution of resistance (Coates et al., 2022). High efficacy therefore improves the long‐term durability of a strategy by reducing the number of rounds of selection. It is, however, important to consider that when the per cent efficacy against susceptible lice is higher, any selection differential also has the potential to be greater. For example, although the efficacy of azamethiphos is close to 100% for susceptible lice, it is much lower for individuals heterozygous and homozygous for the resistant gene (80% and 20%, respectively), resulting in a very strong selection gradient (Myhre Jensen et al., 2017).

### 4.4 Dominance of the resistant allele

The rate at which resistance spread through the metapopulation was influenced by the dominance of the R allele, with our plotted curves matching those expected from evolutionary theory (de Vries et al., 2020; Teshima & Przeworski, 2006). Resistance initially evolved more rapidly with a dominant effect than with an additive effect, due to the higher survival of heterozygotes. It took longer for a dominant R allele to become fixed, however, since the S allele persisted in heterozygotes for a longer time. Conversely, when resistance was recessive, the rise in *p* started slowly but accelerated as the proportion of resistant homozygotes produced increased. Regardless of the strength and shape of the selection gradient, the metapopulation reached a new equilibrium once the R allele was fixed (or lost), which was determined by the fitness of the homozygotes under the treatment strategy (de Vries et al., 2020).

### 4.5 Spatial heterogeneity in the starting frequency of resistance

Our results show that the geographic location at which a resistant mutation arises can have a significant effect on how resistance evolves in the metapopulation. Organophosphate resistance is believed to have been present at a low frequency in the louse population prior to the use of these pesticides on farms (Kaur et al., 2017). By contrast, resistance to pyrethroids and emamectin benzoate likely emerged at single locations and then spread throughout the Atlantic in response to these treatments (Besnier et al., 2014; Fjørtoft et al., 2020). In our model, the main factor influencing the spread of resistance is the location of the mutation relative to the northwards‐flowing Norwegian coastal current. Resistant genes are more likely to become widespread if they arise in the south, as they can ride this current northwards to disperse nationally. Production zones in southern Norway have the highest density of farms (Figure 9), which itself drives rapid adaptation due to high‐transmission rates and concomitantly high‐treatment frequencies (Coates et al., 2022). Our results here add further significance to the Norwegian south‐west (Production Zones 2–4) as an evolutionary hotspot in the salmon network that deserves special attention for monitoring and managing resistance.

### 4.6 Limitations of the model

In our simulations, all farms in the study area imposed the same selection pressure. This greatly increased the overall strength of selection at the metapopulation level for resistance. In reality, there are multiple technologies available to farms which are deployed heterogeneously, thus slowing the rate of evolution to any one treatment (McEwan et al., 2016).

We did not include wild hosts in our simulations, since farmed salmon outnumber wild salmonids in Norway by ~300:1 and so are expected to be the main driver of louse dynamics (Dempster et al., 2021). Nevertheless, a relatively small wild host population could have effects on louse epidemiology and evolution not captured by our model. A population of lice maintained on wild hosts would likely prevent lice from being fully eradicated from the region, despite eradication occurring in our simulations with very high‐treatment efficacy (e.g., the model predicted the extinction of lice after a decade of ubiquitously using a continuous strategy with 80% efficacy). In these scenarios, farms would still become infested, albeit at very low levels, by lice transmitted from wild salmonids. The presence of wild hosts may prevent eradication, but it may also work to slow the evolution of resistance if lice are well controlled on farms. Migrating wild salmonids can act as vectors of louse dispersal, facilitating the movement of genes along different routes to those taken by planktonic larvae (Fjørtoft et al., 2019). Wild hosts receiving lice from farms prior to resistance can act as refugia for susceptible genotypes. This may allow genes for treatment susceptibility to persist in the metapopulation for longer (Bateman et al., 2020; Fjørtoft et al., 2019). Wild host effects are likely to vary spatially and temporally, according to the size and distribution of wild populations over the course of their migration.

Our model assumes that there is no assortative mating of genotypes. Genetic linkage maps suggest that some assortative mating may occur in louse populations, with individuals with similar genetic recombination rates more likely to procreate (Danzmann et al., 2019). However, more data on this are needed before we can incorporate this into the model. Assortative mating of genotypes might occur if resistance is related to other traits (e.g., if lice carrying the R allele produce more attractive chemical cues, or are more active in mate‐searching behaviours). Louse evolutionary dynamics will be different if the recombination of genotypes differs from that expected under the Hardy–Weinberg principle. For example, if individuals with the resistant allele are more likely to mate with one another, this would produce a higher frequency of heterozygous and homozygous resistant offspring than expected. This would accelerate the rate of adaptation. Conversely, negative assortative mating would slow evolution if resistant genotypes are less likely to mate with one another. Note that the model also assumes that males and females – which are not differentiated in the model – do not differ in their expected genotype.

### 4.7 Implications for integrated pest management

We have used this model to make quantitative predictions of louse adaptation which can help inform practical management decisions. Despite our best attempts to parameterise the model with data from the literature, such predictions will always be theoretical to some degree. Model validation becomes increasingly difficult for processes occurring over larger spatial and temporal scales, and when knowledge of the underlying genetics of resistance remains limited (Coates, 2023). Nevertheless, models such as ours lay the groundwork when it comes to developing strategies for integrated pest management. Scenario testing using models informs the efficient design of real‐world trials. More broadly, even abstract attempts to apply evolutionary principles to louse control are better than no attempt at all, especially given the history of resistance repeatedly evolving in the industry (Aaen et al., 2015; Coates, 2023).

One application of the present simulations, for example, is if farms wish to reduce the strength of selection imposed by a certain strategy, by using the treatment less frequently or at a lower dosage. Simulations can estimate the degree to which the selection differential will need to be reduced to slow the spread of resistance by a desired number of years (Figure 5).

Epidemiological models such as ours are useful in estimating the minimum use of a treatment required to keep infestations below acceptable levels, thus reducing any economic, welfare and/or environmental costs associated with the treatment (Moe et al., 2019; Overton et al., 2019). Using a limited number of treatments focused on key farm sites (Samsing et al., 2019) may be the most cost‐effective approach for suppressing lice across the entire region. The efficacy of some louse management strategies is affected by environmental conditions such as temperature and salinity (Coates, Johnsen, et al., 2021; Oldham, 2023). The success of a strategy at controlling lice across a farm network may therefore depend on the location and the season at which it is applied (Barrett, Overton, et al., 2020). In some instances, it may be more cost‐effective to deploy a treatment only where its efficacy exceeds a certain level needed to have an appreciable effect on the metapopulation. Models can thus be a valuable tool for exploring how strategies can be deployed most efficiently in the environment.

Surveillance programs monitoring for resistance (Helgesen et al., 2020) can be guided by model results. For example, our outputs identify areas that act as ‘evolutionary hotspots’ in which monitoring efforts can be focused (Coates et al., 2022). Furthermore, simulations can help researchers decide the sample size needed to detect significant frequencies of resistance in a population. Some types of resistance are predicted to be lost through genetic drift at a low frequency, whilst others (e.g., an allele with high‐relative fitness and an additive effect; Figure 6) can propagate rapidly. For the latter type, a resistant gene will need to be detected at a low frequency in the population, if precautionary action is to be taken before it is too late. This will require taking a larger sample size to be confident that any rare genes will be detected.

A future application of this model will be to explore scenarios where multiple strategies are distributed heterogeneously across farms. Evolutionary models for other systems have shown that a mosaic approach to treatments is highly effective at delaying the evolution of resistance (Onstad et al., 2001, 2013; Rimbaud et al., 2018; Sisterson et al., 2005). Establishing some farms as refugia – i.e., where a pest population is not exposed to a selection pressure – is especially effective if resistant genotypes have reduced fitness in the absence of the treatment (Bateman et al., 2020; Kreitzman et al., 2018). As seen in this study, there is a strong spatial structure to the evolutionary dynamics of lice in the metapopulation. The specific selection pressures experienced at one farm have ripple effects on the infestation pressure and gene flow to neighbouring farms. As such, the exact mosaic pattern will likely determine how successful heterogeneous treatments are at minimising resistance. Which sites should be refugia to slow adaptation? Which sites should combine strategies to control outbreaks? Whilst empirical trials are needed to definitely answer these questions, metapopulation models can provide guidance on how such trials are established most effectively.

Model predictions are most useful for guiding real‐world management decisions when resistant alleles have already been identified, and where there is good data on how they are selected for and inherited. This is the case for azamethiphos resistance. In other cases, the risk of adaptation to a strategy is still theoretical or only supported by correlative evidence (Coates et al., 2020; Coates, Phillips, et al., 2021; Hamre et al., 2021), and we can only estimate how model parameters should be assigned. Targeted research to identify and understand resistance is therefore crucial for each control technology (Coates, 2023). The sooner that data on resistant strains are collected, the sooner that data can be fed into models to predict the evolutionary trajectory of resistance – and ways to mitigate it.

The dynamics explored in these simulations have implications that extend far beyond our study area in Norway. The dispersal of lice on migratory wild salmon is thought to facilitate louse gene flow across the northern Atlantic (Besnier et al., 2014; Boxaspen, 2006). This has previously driven the rapid spread of mutations for resistance from a single location to salmon farms throughout the Atlantic (Besnier et al., 2014; Fjørtoft et al., 2020). Therefore, the factors accelerating adaptation in Norwegian aquaculture, as predicted by our model, would also likely increase the probability of resistance spreading to other salmon farming countries, and vice versa. This highlights the importance of communication and coordination of integrated pest management at an international scale.

Development of this model is an iterative process, and there are many avenues down which this model can be expanded and adapted into the future (e.g., multiple loci for resistance, pleiotropy, heterogeneous distribution of treatments). As this work continues, researchers can continue to sharpen the understanding of louse evolutionary dynamics, and of how to develop integrated pest management that ensures effective parasite control well into the future.

## 5 CONCLUSIONS

We have demonstrated how evolutionary models can not only replicate general evolutionary trends but also make quantitative predictions of how salmon lice may adapt under specific management strategies. These quantitative outputs can be drawn upon during the complex process of making practical management decisions for combatting resistance. Our simulations predicted that continuously acting strategies that increase chalimus mortality were most successful at reducing louse infestations. New and emerging technologies like functional feeds, selective breeding programs, and gene‐edited salmon can control lice in this way (Barrett, Overton, et al., 2020; Robinson et al., 2023).

We identified aspects of resistance that were most conducive to rapid evolution. Lice adapted most quickly when (1) the discrete strategy imposing selection had low‐overall efficacy, (2) a continuous strategy imposed selection on weekly chalimus survival, (3) there was a steep selection gradient across genotypes, (4) resistance was dominant, or (5) resistance initially emerged in the south of Norway. These factors need to be the focus of any directed study into treatment resistance. If these criteria are met, immediate action is needed to limit the evolution of resistance before it becomes widespread.

## FUNDING INFORMATION

This work is part of the project “CrispResist: Harnessing cross‐species variation in sea lice resistance”, funded by the Norwegian Seafood Research Fund (FHF, project 901631).

## CONFLICT OF INTEREST STATEMENT

The authors declare that they have no conflicts of interest.

## Supporting information


Appendix S1.

Figure S1.

## Data Availability

The data and model code used in this article will be shared on reasonable request by the corresponding author.
