# Supplementary material for: Evolutionary predictions for a parasite metapopulation: Modelling salmon louse resistance to pest controls in aquaculture
Source: Evol Appl. 2023 Nov 23;16(12):1982–98. doi: 10.1111/eva.13618 (PMC10739098; doi:10.1111/eva.13618)
Supplement: Supplementary file 1 — Appendix S1. [file EVA-16-1982-s001.docx]

**Supplementary Materials**

This model is an individual-based, stage-structured matrix model that tracks louse numbers over discrete, weekly time-steps, *t*. Our metapopulation was comprised of 537 populations, representing farm sites throughout southern Norway (58.4–66.4° N). The transmission of free-living larvae between farms was parameterised using particle-tracking model outputs produced for this region by Samsing et al., (2017).

Each farm, *i*, was assigned a mean weekly temperature, *T_ti_* (°C), for each corresponding time-step*, t*. This was to allow for spatial and temporal differences in louse development due to temperature variation (Hamre et al., 2019). Mean temperatures were calculated over five-week periods, using the data available from barentswatch.no for each farm (Coates et al., 2022). The number of hosts per farm was calculated from the maximum allowed biomass for each farm (as provided by Samsing et al., 2017), divided by the mean weight of salmon at harvest (4.5 kg; Barrett et al., 2022).

Lice were grouped into the four major stages of the parasite’s life cycle (Hamre et al., 2019): ‘larva’, ‘chalimus’, ‘pre-adult’ and ‘adult’, notated here as $b=\left\{ L, C, P, A \right\},$ $b=\left\{ L, C, P, A \right\},$ respectively. At each time-step, a proportion of lice progressed to the subsequent life stage. Adult lice remained in the adult stage and produced new larvae every *t*.

For a single farm, the number of lice at *t* is given by the column vector **N**, where each row corresponds to the number of lice (*n*) of each life stage. Assuming just one genotype (*g*), the number of lice at *t+1* is given by **N** at the previous time-step, multiplied by a transition matrix, **S**:

$\mathbf{N}_{t+1,g}=\mathbf{S}_{g}\cdot\mathbf{N}_{\mathrm{tg}}=\left[ \begin{matrix} 0 & 0 & 0 & fu_{g} \\ dv & \left( 1-\delta\right)*(1-\mu_{C}) & 0 & 0 \\ 0 & \delta*(1-\mu_{C}) & \left( 1-\delta\right)*(1-\mu_{P}) & 0 \\ 0 & 0 & \delta*(1-\mu_{P}) & 1-\mu_{A} \end{matrix} \right]\cdot\left[ \begin{matrix} n_{L} \\ n_{C} \\ n_{P} \\ n_{A} \end{matrix} \right]_{\mathrm{tg}}$ (1)

The parameter *δ* is the proportion of lice that develop to the next life stage over one week, and (1 – *δ*) is the proportion remaining in the current stage. The value of *δ* is calculated according to the temperature, *T* (°C), on the farm at *t*. We used the equation given in Hamre *et al.*, (2019) to calculate the daily transition rate of lice (Table 1). The constants are those provided by Hamre et al., (2019) averaged across males and females, since sexes were not differentiated in our model. The daily transition rate was then converted into the weekly rate (Table 1).

A proportion of lice, *µ*, was also lost each week through a background mortality rate (Figure 1). We used the lower range of daily mortality rates estimated by Stien et al., (2005), averaged across sexes: 0.002 individual^-1^ day^-1^ for chalimi, and 0.025 individual^-1^ day^-1^ for pre-adults and adults. These were converted into weekly background mortality rates for chalimi, pre-adults and adults; *µ_C_*, *µ_P_* and *µ_A_*, respectively (Table 1). The background mortality of larvae was already accounted for in the larval dispersal parameter *d* (see below).

The mean number of larvae produced per adult per week, *f*, was determined by the temperature at the corresponding farm and time-step. We used the equations given in Johnsen et al., (2020) to calculate the number of eggs per egg-string (*N_eggs_*) and the number of days between clutches of egg-strings hatching (*D_hatch_*) at temperature *T* (Table 1). Females produce one pair of egg-strings per clutch. Given a 1:1 sex ratio, this means an average of one egg-string is produced per adult at a time. The daily production of larvae per adult was therefore calculated as N_eggs_/D_hatch_, which was multiplied by seven for a weekly larval output per adult (Table 1).

The parameter *u_g_* in Equation 1 is the proportion of larvae produced that carry the genotype, *g* (in this example with one ubiquitous genotype, *u*_g_ = 1).

Of the larvae produced on the farm at *t*, a proportion, *d*, returns to the farm at *t+1* as an infective copepodid. The value of *d* (here, the probability of ‘self-recruitment’) for any given farm was provided by particle-tracking model outputs (Samsing et al., 2017). Of the copepodids that reach a farm site, a proportion, *v*, successfully re-enter a cage and attach to a host. There are no empirically-determined estimates for *v*. Previously, Coates et al., (2022) parameterised *v* = 0.05 after comparing model predictions with reported farm infestations (barentswatch.no). More recently, in a different salmon louse metapopulation model, Kragesteen et al., (2023) estimated louse attachment rates as a function of the ratio between the volume of salmon cages, and the total volume of the farm site used by the particle-tracking model. In that study, the proportion of incoming copepodids that reached a host (*v* in our notation) was calculated as:

$v= \sqrt{\frac{W}{\rho V}}$ (2)

where *W* is the biomass (kg) of the farm, *ρ* is the stocking density of the farm (assumed to be 20 kg/m^3^), and *V* is the total volume (*m^3^*) of the farm site in the particle-tracking model that received incoming lice. The particle-tracking model that we have used for our simulations encompasses a much larger geographical area than that in Kragesteen *et al.*, (2023), but at the compromise of having a much coarser spatial resolution. As such, the total volume of a farm site, *V*, was larger in our model (*V* = 2400 * 2400 * 15 m) than that used in Kragesteen et al., (2023). Inputting our *V* value and our mean farm biomass (*W* = 3000 t) into Eq. 2, produces *v* = 0.042. Pleasingly, this is in accordance with our initial estimate of 0.05. Given the relatively large farm areas in our particle-tracking model and the limited knowledge on fine-scale copepodid dispersal, we have kept *v* = 0.05 constant across farms (Table 1).

Lice were grouped according to genotype, *g*, at a single, biallelic locus. Genotypes are notated here as $g=\{RR, RS, SS\}$, where *R* is the resistant (mutant) allele and *S* the susceptible (wild-type) allele. The number of larvae of each genotype produced was calculated according to the Hardy-Weinberg principle: the expected proportions of RR, RS and SS offspring are *p*^2^, 2*pq* and *q*^2^, respectively, where *p* and *q* are the frequencies of the R and S alleles in the parent population (Falconer and Mackay, 1996).

To capture multiple genotypes, Equation 1 was expanded to block matrix form as:

$\mathbf{L}_{t+1}=\mathbf{G}\cdot\mathbf{L}_{t}=\left[ \begin{matrix} \mathbf{S}_{\mathrm{RR}} & \mathbf{F}_{\mathrm{RR}} & \mathbf{F}_{\mathrm{RR}} \\ \mathbf{F}_{\mathrm{RS}} & \mathbf{S}_{\mathrm{RS}} & \mathbf{F}_{\mathrm{RS}} \\ \mathbf{F}_{\mathrm{SS}} & \mathbf{F}_{\mathrm{SS}} & \mathbf{S}_{\mathrm{SS}} \end{matrix} \right]\cdot\left[ \begin{matrix} \mathbf{N}_{\mathrm{RR}} \\ \mathbf{N}_{\mathrm{RS}} \\ \mathbf{N}_{\mathrm{SS}} \end{matrix} \right]_{t}$ (3)

where **L** is the number of lice across all life stages and genotypes, and **G** is the matrix for stage and genotype transitions. Within **G**, the matrices **S_g_** are the same as in Equation 1, but with *u*_g_ equal to either *p*^2^, 2*pq* or *q*^2^; for **S**_RR_, **S**_RS_, and **S**_SS_, respectively. New values for *p* and *q* (allele frequencies in the adult population) are calculated at each time-step. The matrices **F_g_** within **G** are:

$\mathbf{F}_{g}=\left[ \begin{matrix} 0 & 0 & 0 & fu_{g} \\ 0 & 0 & 0 & 0 \\ 0 & 0 & 0 & 0 \\ 0 & 0 & 0 & 0 \end{matrix} \right]$ (4)

again where *u*_RR_, *u*_RS_ and *u*_SS_ is *p*^2^, 2*pq* and *q*^2^, respectively.

The single-farm Eq. 3 was nested within a matrix model for multiple farm sites, given by:

$\mathbf{P}_{t+1}=\mathbf{M}\cdot\mathbf{P}_{t}$ (5)

where **P** is the louse metapopulation, composed of the louse populations, **L**, at each farm, *i*:

$\mathbf{P}_{t}=\left[ \begin{matrix} \mathbf{L}_{1} \\ \mathbf{L}_{2} \\ \vdots\\ \mathbf{L}_{i} \end{matrix} \right]_{t}$(6)

and **M** is the block matrix:

$\mathbf{M}=\left[ \begin{matrix} \mathbf{G}_{1} & \mathbf{C}_{21} & \cdots& \mathbf{C}_{i1} \\ \mathbf{C}_{12} & \mathbf{G}_{2} & \cdots& \mathbf{C}_{i2} \\ \vdots& \vdots& \ddots& \vdots\\ \mathbf{C}_{1i} & \mathbf{C}_{21} & \cdots& \mathbf{G}_{i} \end{matrix} \right]$ (7)

with the number of rows and columns equal to the number of farms in the simulation. On the leading diagonal of **M** is the transition matrix **G**_i_ for each farm. These capture within-farm processes (survival, development, reproduction and larval self-recruitment). The matrices **G**_i_ are equal to **G** in Eq. 3, but with unique self-recruitment values (*d*) for each farm: *d*_ii_, which is the probability of dispersing from farm *i* back to farm *i*. Unique gene frequency values (*p* and *q*) are also calculated for each farm site, according to the total number of adults at that site.

The rest of **M** contains connectivity matrices, **C**_ji_, which represent the probability of larval dispersal from farm *j* (corresponding to columns in **M**) to farm *i* (corresponding to rows in **M**). The matrix **C**_ji_ is given by:

$\mathbf{C}_{\mathrm{ji}}=\left[ \begin{matrix} \mathbf{D}_{RR} & \mathbf{Z} & \mathbf{Z} \\ \mathbf{Z} & \mathbf{D}_{RS} & \mathbf{Z} \\ \mathbf{Z} & \mathbf{Z} & \mathbf{D}_{SS} \end{matrix} \right]_{\mathrm{ji}}$(8)

and where **D**_g_ is:

$\mathbf{D}_{g}=\left[ \begin{matrix} 0 & 0 & 0 & 0 \\ d_{ji}v & 0 & 0 & 0 \\ 0 & 0 & 0 & 0 \\ 0 & 0 & 0 & 0 \end{matrix} \right]_{g}$(9)

**D**_g_ contains *d*_ji_, which is the probability of larvae (of genotype *g*) dispersing from farm *j* to farm *i*. The rest of **C**_ji_ is filled with **Z,** which represents a 4 x 4 matrix containing zeros (as larval dispersal of one genotype does not influence recruitment of the other genotypes).

Every combination of *d_ji_* was assigned values from the Samsing et al., (2017) particle-tracking model outputs. Dispersal probabilities were simulated over two periods: November–February, and April–July. We used the April–July *d*_ji_ values for weeks 11–38 in our simulation, and the November–February values for the remaining weeks. Dispersal probabilities for each season were averaged over the 6 years (2009–2014) that were simulated by Samsing et al., (2017).

In scenarios using a discrete management strategy, delousing occurred when the adult louse abundance on a farm (the number of adults / the number of hosts) exceeded a treatment threshold, *k*. We assigned *k* = 1 adult louse fish^-1^, or *k* = 0.4 adult louse fish^-1^ during weeks 16–21 of the year. These correspond to Norway’s legal lice limits of 0.5 adult females fish^-1^, or 0.2 adult females fish^-1^ in spring (Sandvik et al., 2021), assuming a 1:1 sex ratio in adults. These limits most commonly precipitate delousing events, as seen in the barentswatch.no database (Coates et al., 2022).

Treatments were applied by multiplying louse numbers by (1 – *x_bg_*), immediately after calculating Eq. 5 in the model. The parameter *x* represents delousing mortality (i.e. treatment efficacy) and which could be assigned unique values for each life stage (*b*) and genotype (*g*).

As in Coates et al., (2022) we also included a ‘forced harvest’ of farms (resulting in 100% mortality of all attached lice) when adult abundance exceeded 2 adults fish^-1^. This was included to reflect the harvesting of farms in response to higher infestations (as seen in the barentswatch.no farm records), to account for harvesting as part of the regular farm production cycle, and to dampen any unreasonably high infestations after widespread evolution of resistance (since our farms could not turn to alternative management strategies in these simulations).

Farms could also be assigned continuously-acting management strategy with various lethal or sublethal effects. In these scenarios, the model parameters *v*, *µ_C_*, *δ,* or *f* in the transition matrix **M** could be adjusted by *y_v_*, *y_µ_*, *y_δ_* or *y_f_*, respectively. The values for *y* are the proportional reductions (i.e., treatment efficacy) in the related parameter. Different *y* values could be assigned for each genotype.

The model was constructed and run on R version 4.0.

**References**

Barrett, L., Oldham, T., Kristiansen, T.S., Oppedal, F., Stien, L.H., 2022. Declining size-at-harvest in Norwegian salmon aquaculture: lice, disease, and the role of stunboats. Aquaculture 559, 738440. https://doi.org/10.1016/j.aquaculture.2022.738440

Falconer, D., Mackay, T., 1996. Introduction to quantitative genetics, 4th ed. ed. Longman, Essex, England.

Hamre, L., Bui, S., Oppedal, F., Skern-Mauritzen, R., Dalvin, S., 2019. Development of the salmon louse Lepeophtheirus salmonis parasitic stages in temperatures ranging from 3 to 24°C. Aquac. Environ. Interact. 11, 429–443. https://doi.org/10.3354/aei00320

Johnsen, I., Stien, L., Sandvik, A., Asplin, L., Oppedal, F., 2020. Optimal estimation of lice release from aquaculture based on ambient temperatures. Aquac. Environ. Interact. 12, 179–191. https://doi.org/10.3354/aei00358

Kragesteen, T.J., Johannesen, T.T., Sandvik, A., Andersen, K.H., Johnsen, I.A., 2023. Salmon lice dispersal and population model for management strategy evaluation. Aquaculture 575. https://doi.org/10.1016/j.aquaculture.2023.739759

Sandvik, A.D., Bui, S., Huserbråten, M., Karlsen, Ø., Myksvoll, M.S., Ådlandsvik, B., Johnsen, I.A., 2021. The development of a sustainability assessment indicator and its response to management changes as derived from salmon lice dispersal modelling. ICES J. Mar. Sci. 78, 1781–1792. https://doi.org/10.1093/icesjms/fsab077

Stien, A., Bjørn, P.A., Heuch, P.A., Elston, D.A., 2005. Population dynamics of salmon lice Lepeophtheirus salmonis on Atlantic salmon and sea trout. Mar. Ecol. Prog. Ser. 290, 263–275. https://doi.org/10.3354/meps290263
