# Supplementary figures and images for: Evolutionary predictions for a parasite metapopulation: Modelling salmon louse resistance to pest controls in aquaculture
Source: Evol Appl. 2023 Nov 23;16(12):1982–98. doi: 10.1111/eva.13618 (PMC10739098; doi:10.1111/eva.13618)

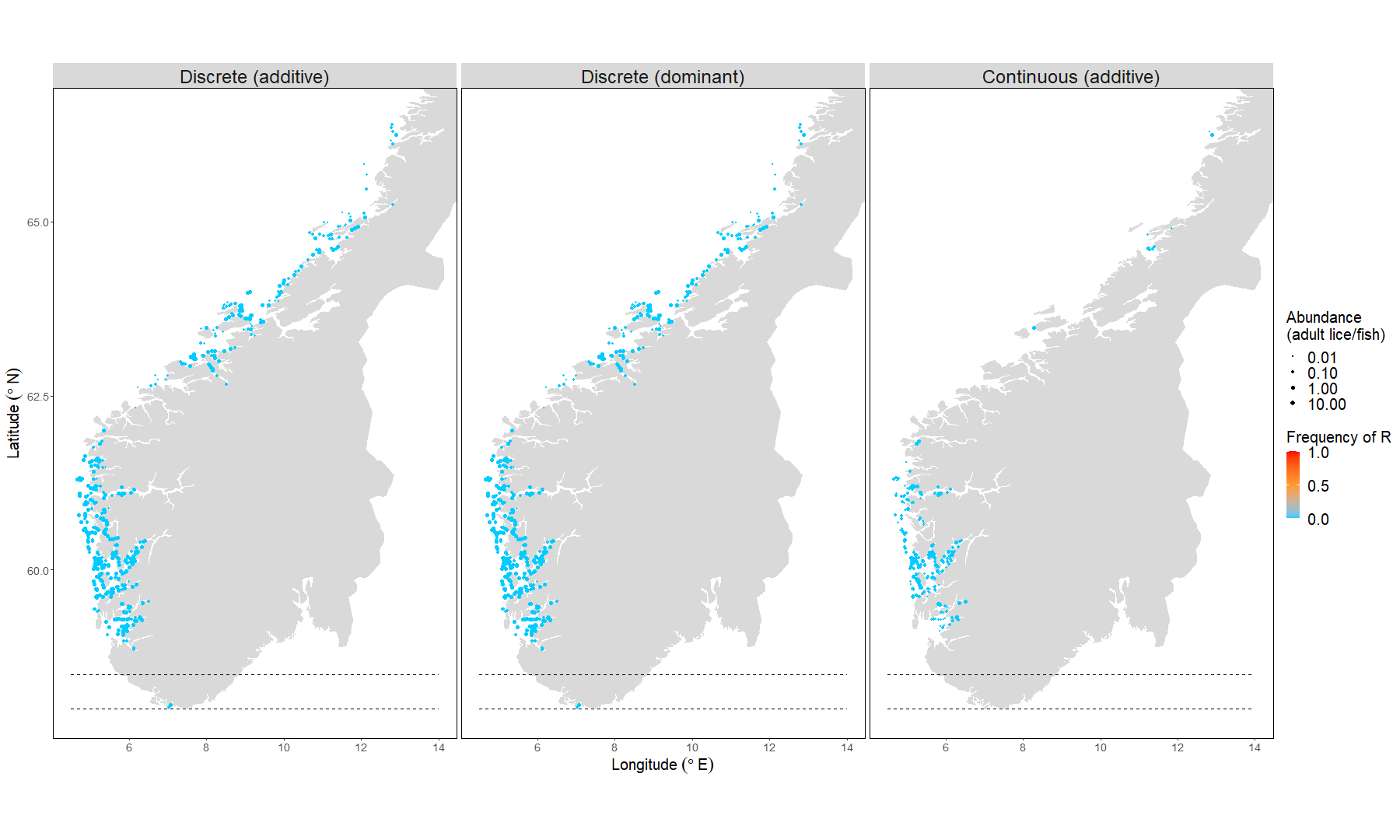

Supplement: Supplementary file 2 — Figure S1. [file EVA-16-1982-s002.gif]
